# Enhanced glucose metabolism through activation of HIF-1α covers the energy demand in a rat embryonic heart primordium after heartbeat initiation

**DOI:** 10.1038/s41598-021-03832-5

**Published:** 2022-01-07

**Authors:** Tatsuya Sato, Nobutoshi Ichise, Takeshi Kobayashi, Hiroyori Fusagawa, Hiroya Yamazaki, Taiki Kudo, Noritsugu Tohse

**Affiliations:** 1 Department of Cellular Physiology and Signal Transduction, Sapporo Medical University School of Medicine, South-1, West-17, Chuo-ku, Sapporo, 060-8556 Japan; 2 Department of Cardiovascular, Renal and Metabolic Medicine, Sapporo Medical University School of Medicine, Sapporo, Japan; 3 Department of Orthopedic Surgery, Sapporo Medical University School of Medicine, Sapporo, Japan

**Keywords:** Developmental biology, Physiology, Cardiology

## Abstract

The initiation of heartbeat is an essential step in cardiogenesis in the heart primordium, but it remains unclear how intracellular metabolism responds to increased energy demands after heartbeat initiation. In this study, embryos in Wistar rats at embryonic day 10, at which heartbeat begins in rats, were divided into two groups by the heart primordium before and after heartbeat initiation and their metabolic characteristics were assessed. Metabolome analysis revealed that increased levels of ATP, a main product of glucose catabolism, and reduced glutathione, a by-product of the pentose phosphate pathway, were the major determinants in the heart primordium after heartbeat initiation. Glycolytic capacity and ATP synthesis-linked mitochondrial respiration were significantly increased, but subunits in complexes of mitochondrial oxidative phosphorylation were not upregulated in the heart primordium after heartbeat initiation. Hypoxia-inducible factor (HIF)-1α was activated and a glucose transporter and rate-limiting enzymes of the glycolytic and pentose phosphate pathways, which are HIF-1α-downstream targets, were upregulated in the heart primordium after heartbeat initiation. These results suggest that the HIF-1α-mediated enhancement of glycolysis with activation of the pentose phosphate pathway, potentially leading to antioxidant defense and nucleotide biosynthesis, covers the increased energy demand in the beating and developing heart primordium.

## Introduction

The heart in an embryo, which plays a central role in fetal circulation, develops and functions ahead of other organs^1^. Therefore, functional adaptations as well as morphological changes in the early embryonic heart in a vertebrate are essential for normal fetal growth and development. The heart primordium that will form the future embryonic heart arises as populations of cardiac progenitor cells derived from the lateral plate mesoderm in a crescent shape in mice^2^ and rats^3^ and in a bilateral pair in humans^4^ and birds^5^. We previously reported that heartbeat in a rat embryonic heart primordium is initiated at embryonic day 9.99–10.13 (E9.99–E10.13) with a calcium transient via extracellular calcium influx preceding muscle contraction^6^. Although the chronological timeline of developmental stages and concomitant heartbeat initiation timing have reported to be different depending on the species^5–9^, the embryonic heart primordium after heartbeat initiation requires energy for driving the excitation–contraction coupling. Hence, in the heart primordium in the period around the initial heartbeat, energy metabolism has to be altered for adapting to produce more ATP not only for differentiation and proliferation but also for maintenance of heartbeat, but it remains unclear how energy metabolism responds to increased energy demand.

Glycolysis has been reported to be a major source of energy in mesodermal and cardiac progenitor cells during early cardiac development^10–12^. The oxygen supply to the embryo is limited to diffusion from the placenta until fetal circulation is formed, and thus it is reasonable that glycolysis, which does not require oxygen to produce ATP, plays a central role in energy production in the heart primordium. In contrast, it has been acknowledged that mitochondrial oxidative phosphorylation (OXPHOS), which can produce large amounts of ATP compared with those produced by anerobic glycolysis under the condition of a sufficient supply of oxygen and energy substrates, contributes less to energy metabolism in the embryonic heart than that in the matured heart^10–12^. In fact, cristae density and the number of mitochondria in cardiomyocytes have been reported to be smaller in the embryonic heart than in the matured heart^12–14^, suggesting less reliance on mitochondrial OXPHOS for the production of ATP in cardiac progenitor cells or cardiomyocytes in the embryo. However, it remains unclear whether mitochondria play any roles in response to increased energy demand to initiate excitation–contraction coupling in the developing heart primordium. It is also unknown whether oxygen consumption with or without activation of mitochondrial OXPHOS itself is increased to respond to increased energy demand in the heart primordium after heartbeat initiation.

In terms of oxygen sensing, hypoxia-inducible factor (HIF) pathways are known to play a central role in response to hypoxia^15^. HIF is a transcriptional factor that is induced in response to hypoxia and regulates the expression of hypoxia-responsive genes. The embryo develops in a low oxygen environment, and activation of HIF pathways has been shown to be essential for placental formation, vasculogenesis, and cardiac development^16–18^. There are three HIF-α subunits (HIF-1α, HIF-2α, and HIF-3α) and, indeed, it has been reported that loss of HIF-1α in mice showed various phenotypes in impaired cardiogenesis including morphogenesis arrest, reduced endothelial cells, myocardial hyperplasia, and impaired neural cell migration at the early stages of embryonic development^19–21^. In association with HIF pathways and energy metabolism, it has been reported that activation of HIF-1α pathways transcriptionally upregulates glucose transporters, rate-limiting enzymes in the glycolytic pathway and the pentose phosphate pathway, and pyruvate dehydrogenase kinase-1 (PDK1), leading to inactivation of pyruvate dehydrogenase (PDH) complex in the tricarboxylic acid (TCA) cycle^22–24^. These regulations potentially result in enhanced glycolysis, increased activity of the pentose phosphate pathway, and decreased mitochondrial respiration. Thus, HIF-1 pathways must play an important role in energy metabolism in the developing heart primordium, yet the details of HIF-1α-mediated metabolic alteration at the period of initial heartbeat in the heart primordium have not been elucidated.

Therefore, in the present study, we examined how energy metabolism in the embryonic heart is altered after the initiation of heartbeat by performing unbiased metabolomic analysis and evaluating mitochondrial function and glycolysis using an extracellular flux analyzer. We also investigated whether HIF-1α is activated in response to increased energy demand and whether expression of its downstream targets in glycolysis, pentose phosphate pathway, and TCA cycle is altered in the heart primordium after heartbeat initiation compared with that before heartbeat initiation.

## Results

### Definitions of pre- and post-heartbeat groups

**Figure 1:**
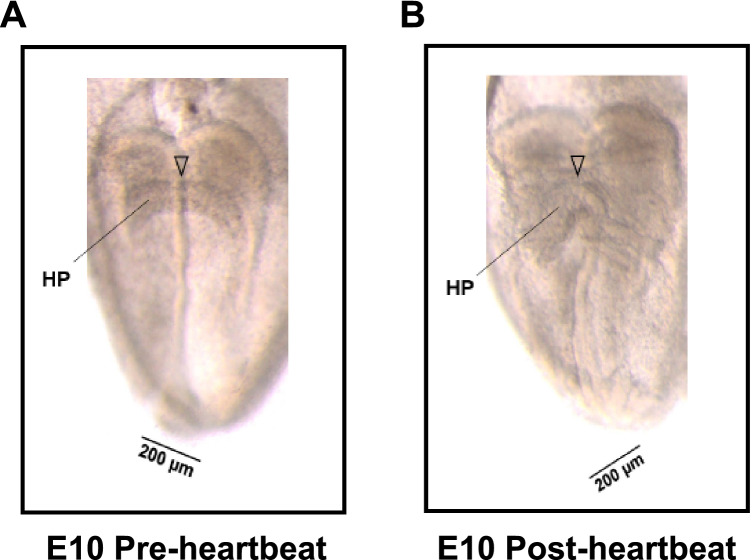
Definitions of rat heart primordia before (pre-) and after (post-) heartbeat initiation. (**A**) Representative image of a rat heart primordium in the embryo at E10 before (pre-) heartbeat initiation. (**B**) Representative image of a rat heart primordium in the embryo at E10 after (post-) heartbeat initiation. The arrowhead indicates the center of the heart primordia. We defined heart primordia showing a “flat” central shape as the pre-heartbeat group and heart primordia showing a “raised” central shape as the post-heartbeat group according to the findings in a previous study^6^. HP: heart primordium.

In accordance with our previous study^6^ showing that there is a relationship between morphologic characteristics and timing of the initiation of contraction in the heart primordium at E10, we defined heart primordia showing a “flat” central shape in embryos with three to four somites as the pre-heartbeat group (heart primordia before heartbeat initiation) and heart primordia showing a “raised” central shape in embryos with four to five somites as the post-heartbeat group (heart primordia after heartbeat initiation) in the present study (Fig. 1).

### Metabolomic analysis in the heart primordium before and after heartbeat initiation

First, we performed metabolome analysis to assess how metabolites in the heart primordium are altered after heartbeat initiation. Since very small amount of metabolites was expected in a single heart primordium, six isolated heart primordia in the same group were pooled as one sample, and three samples each in the pre-heartbeat group and the post-heartbeat group were used for metabolome analysis. Of the 116 metabolites that are measurable by capillary electrophoresis time-of-flight mass spectrometry (CE-TOFMS) and capillary electrophoresis tandem mass spectrometry (CE-MS/MS), 63 metabolites were detected in the heart primordium (Supplementary Table S1).

**Figure 2:**
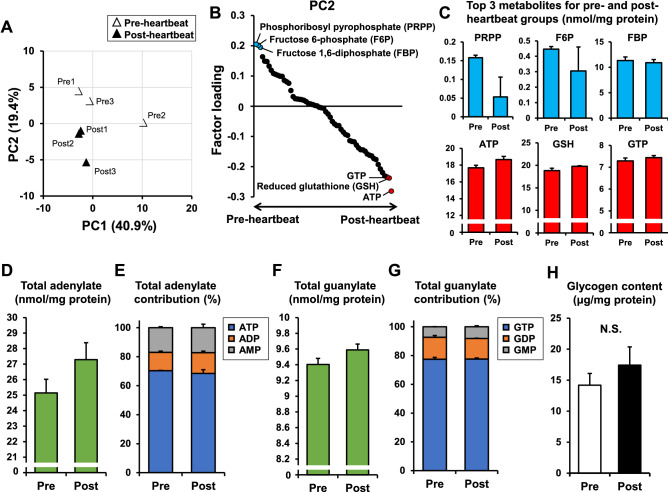
Characteristics of energy metabolites in the heart primordium before and after heartbeat initiation. (**A**) Score plot of principal component analysis in the metabolome analysis. (**B**) Factor loading plot for PC2. Metabolites detected in the metabolome analysis are arranged on the X-axis in the order of factor loading. Blue dots indicate the top three metabolites showing positive factor loading and red dots indicate top three metabolites showing negative factor loading. (**C**) Individual values of the top three metabolites for the pre-heartbeat group (PRPP, F6P, and FBP) and for the post-heartbeat group (ATP, GSH, and GTP). (**D**,**E**) Total adenylate and its contribution that were obtained by metabolome analysis in the pre- and post-heartbeat groups. (**F**,**G**) Total guanylate and its contribution that were obtained by metabolome analysis in the pre- and post-heartbeat groups. (**H**) Glycogen contents assessed by a colorimetric glycogen assay in the pre- and post-heartbeat groups (N = 6). *p < 0.05 with Welch's t-test. N.S.: not significant.

Principal component analysis using the metabolites detected by CE-TOFMS and CE-MS/MS was performed to focus on the metabolites that characterize the difference between the pre- and post-heartbeat groups. A score plot of the top two components in principal component analysis revealed that the second component (PC2), which accounted for 19.4% of the total variation, clearly separated the post-heartbeat group from the pre-heartbeat group, while the first component (PC1) appeared to be affected by the sample including an outlier (Fig. 2A). A factor loading plot for PC2 revealed that phosphoribosyl pyrophosphate (PRPP), fructose 6-phosphate (F6P), and fructose 1,6-bisphosphate (FBP) were the top three metabolites contributing more to the total variance for the pre-heartbeat group, whereas ATP, reduced glutathione (GSH), and GTP in that order were the top three metabolites contributing more to the total variance for the post-heartbeat group (Fig. 2B,C).

The results showing that the increased level of ATP, a major product of glucose catabolism, was the best determinant factor in the post-heartbeat group compared with the pre-heartbeat group indicate that energy production was preferentially increased in the heart primordium after heartbeat initiation (Fig. 2B). Since GSH is produced by glutathione reductase from its oxidized form glutamine disulfide (GSSG) depending on nicotinamide adenine dinucleotide phosphate (NADPH) in the oxidative stage of the pentose phosphate pathway^25^, it is likely that the pentose phosphate pathway was activated in the heart primordium after heartbeat initiation (Fig. 2B). In fact, the finding that F6P and FBP, which are intermediate metabolites in the glycolytic pathway, were the major determinants in the pre-heartbeat group may reflect changes in glucose flow from canonical glycolysis to the pentose phosphate pathway in the heart primordium after heartbeat initiation (Fig. 2B). In addition to these pathways, the purine nucleotide synthesis pathway is likely to be activated in the heart primordium after the initiation of heartbeat because not only ATP but also GTP, which is the other end-product in purine nucleotide synthesis, were the major contributing factors in the post-heartbeat group. The finding that PRPP, which is an intermediate metabolite in the purine and pyrimidine nucleotide synthesis pathway^26^, was the most contributing factor in the pre-heartbeat group also supports the possibility that PRPP is utilized for nucleotide synthesis in the heart primordium after heartbeat initiation (Fig. 2B). Indeed, the level of total adenylate, which is defined as the sum of ATP, ADP and AMP, and the level of total guanylate, which is defined as the sum of GTP, GDP and GMP, were considerably higher in the post-heartbeat group than in the pre-heartbeat group (Fig. 2D,F), though statistical comparisons were not performed in each metabolite that was obtained from multi-omics metabolome analysis to avoid the limitation of sample size and multiple testing. For total adenylate, the distributions of ATP, ADP and AMP in the pre-heartbeat group were 70.3%, 12.8%, and 16.9%, respectively, and those distributions in the post-heartbeat group were 68.4%, 14.3%, 17.3%, respectively (Fig. 2E). Given the finding that an increased level of ATP is the most contributing factor for the post-heartbeat group (Fig. 2B, C), the data possibly suggest that ATP hydrolysis increases after the initiation of heartbeat and its increased energy demand is met by ATP synthesis and the activated purine synthesis pathway. In contrast to total adenylate, the distributions of GTP, GDP, and GMP were similar in the pre- and post-heartbeat groups (Fig. 2G). There was no statistical difference in the glycogen contents that were obtained by a colorimetric assay in the pre- and post-heartbeat groups (Fig. 2H), suggesting that glycogen is not a major carbon source in response to increased energy demand after heartbeat initiation.

In the metabolome analysis, the role of mitochondria in the increased ATP level in the post-heartbeat group was not uncovered because lactate, which is the end-product of anaerobic glycolysis, and metabolites associated with the TCA cycle and mitochondrial OXHPOS were not highlighted (Fig. 2B).

### Assessment of mitochondrial respiration and glycolytic ability in the heart primordium before and after heartbeat initiation

**Figure 3:**
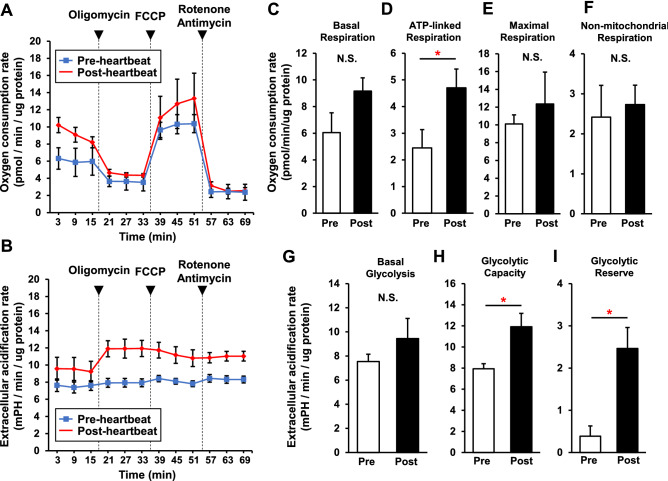
Increased glycolytic capacity and ATP synthesis-linked mitochondrial respiration in the heart primordium after heartbeat initiation. Analysis of OCR and ECAR was carried out using a Seahorse XFe96 Bioanalyzer to assess mitochondrial respiration and glycolysis. (**A**,**B**) OCR (**A**) and ECAR (**B**) were measured in isolated cells from the heart primordium in the pre- and post-heartbeat groups (N = 5) with subsequent addition of oligomycin (complex V inhibitor), FCCP (a protonophore), and rotenone (complex I inhibitor)/antimycin A (complex III inhibitor). (**C**) Mitochondrial bioenergetic parameters calculated from the results for OCR in the pre- and post-heartbeat groups. (**D**) Glycolytic parameters calculated from the results for ECAR in the pre- and post-heartbeat groups. *p < 0.05 with Welch's t-test. N.S.: not significant.

Oxygen consumption rate (OCR) and extracellular acidification rate (ECAR) in isolated cells from the heart primordia were simultaneously measured in a real-time manner to assess mitochondrial respiration and glycolytic ability (Fig. 3A,B).

Basal OCRs were statistically comparable in the pre- and post-heartbeat groups (6.05 ± 1.48 vs. 9.16 ± 0.99 pmol/min/μg protein) (Fig. 3C). However, ATP-linked respiration, which is derived from the difference between basal OCR and respiration following addition of the complex V inhibitor oligomycin, was significantly higher in the post-heartbeat group (4.70 ± 0.70 pmol/min/μg protein) than in the pre-heartbeat group (2.45 ± 0.69 pmol/min/μg protein), suggesting that oxygen consumption for ATP production in the mitochondria was increased in the heart primordium after heartbeat initiation (Fig. 3D). Despite the increased ATP-linked respiration, the levels of maximal respiration induced by carbonyl cyanide p-trifluoromethoxyphenylhydrazone (FCCP), a protonophore, were not significantly different in the pre- and post-heartbeat groups (10.11 ± 1.02 vs. 12.36 ± 3.58 pmol/min/μg protein), presumably due to the immature mitochondrial OXPHOS system in both the pre- and post-heartbeat groups (Fig. 3E). There was no statistical difference in non-mitochondrial respiration between the pre- and post-heartbeat groups (2.42 ± 0.79 vs. 2.73 ± 0.48 pmol/min/μg protein) (Fig. 3F).

Similar to basal OCR, ECAR at baseline, which reflects glycolysis in the presence of oxygen, in the pre-heartbeat group (7.54 ± 0.61 mPH/min/μg protein) was comparable to that in the post-heartbeat group (9.45 ± 1.66 mPH/min/μg protein) (Fig. 3G). However, ECAR in the presence of oligomycin, which reflects glycolytic capacity, was significantly higher in the post-heartbeat group (11.91 ± 1.27 mPH/min/μg protein) than in the pre-heartbeat group (7.93 ± 0.49 mPH/min/μg protein), suggesting that the rate of glucose catabolism was facilitated independently of oxygen consumption in the mitochondria in the heart primordium after heartbeat initiation (Fig. 3H). Furthermore, glycolytic reserve, which is defined as the difference between basal ECAR and ECAR following oligomycin addition, was also significantly higher in the post-heartbeat group (2.46 ± 0.50 mPH/min/μg protein) than in the pre-heartbeat group (0.39 ± 0.24 mPH/min/μg protein), indicating a higher capability of glycolysis to respond to an energy demand in the heart primordium after heartbeat initiation (Fig. 3I).

These results demonstrate that increased glycolytic capacity, rather than activation of mitochondrial OXPHOS itself, mainly contributes to increased ATP production in the heart primordium after heartbeat initiation.

### Activated HIF-1α with its increased protein expression in the heart primordium after heartbeat initiation

**Figure 4:**
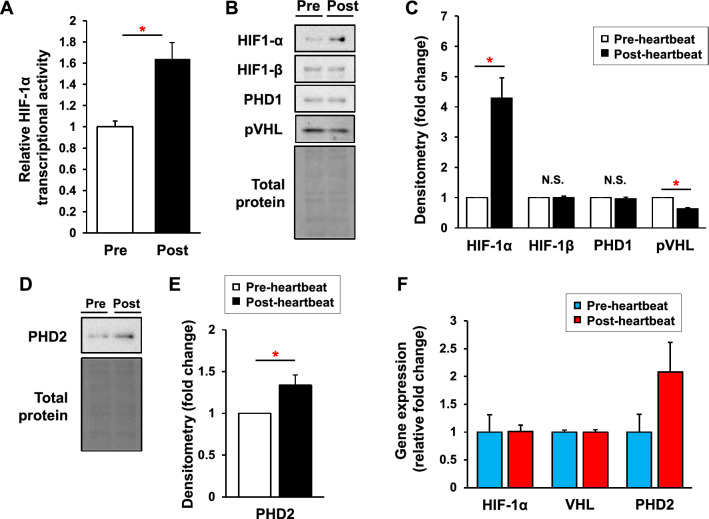
Activated HIF-1α with its increased protein expression in the heart primordium after heartbeat initiation. (**A**) DNA-binding activity of HIF-1α in the nuclear fraction in the pre- and post-heartbeat groups (N = 6). (**B**,**C**) Representative images of Western blots (**B**) and densitometry (**C**) of HIF-1α, HIF-1β, PHD1, and pVHL in the pre- and post-heartbeat group (N = 5–6). (**D**,**E**) Representative images of Western blots (**D**) and densitometry (**E**) of PHD2 in the pre- and post-heartbeat groups (N = 6). (**F**) Relative gene expression levels of HIF-1α, VHL, and PHD2 that were obtained by microarray analysis in the pre- and post-heartbeat groups. *p < 0.05 with Welch's t-test. N.S.: not significant. Full-length blots/gels are presented in Supplementary Figure S1.

We addressed the underlying molecular mechanisms in which glycolytic capacity is increased in the heart primordium after heartbeat initiation. Since HIF-1α is one of the strongest positive regulators for the glycolytic pathway^22^, we examined the DNA-binding activity of HIF-1α. As expected, DNA-binding activity of HIF-1α in the nuclear fraction of the heart primordium was significantly higher in the post-heartbeat group than in the pre-heartbeat group (Fig. 4A). Being consistent with its activity, the protein expression level of HIF-1α was significantly higher in the post-heartbeat group than in the pre-heartbeat group (Fig. 4B,C). Protein levels of HIF-1β, an obligatory dimerization partner for HIF-1α, and prolyl hydroxylase-1 (PHD1), an oxygen-sensing protein that is not regulated by HIF-1α and controls degradation of α-subunits of HIFs via hydroxylation^27^, were comparable in the pre- and post-heartbeat groups, whereas the von Hippel–Lindau protein (pVHL), a substrate recognition component of an E3-ubiquitin ligase that ubiquitylates HIF-1α, was significantly decreased in the post-heartbeat group compared with that in the pre-heartbeat group (Fig. 4B,C). These findings suggest that HIF-1α is upregulated in the heart primordium after heartbeat initiation with reduced pVHL expression presumably contributing to reduction of HIF-1α ubiquitination and degradation.

We further assessed the protein level of PHD2, another oxygen-sensing protein that negatively controls α-subunits of HIFs and is also regulated by HIF-1α itself^27^. The PHD2 protein level was significantly increased in the post-heartbeat group compared with that in the pre-heartbeat group (Fig. 4D,E), supporting our finding that the HIF-1α pathway is actually activated in the heart primordium after heartbeat initiation. Considering that both the activity and the protein level of HIF-1α were increased in the heart primordium after heartbeat initiation (Fig. 4A–C), increased protein level of PHD2 in the heart primordium after heartbeat initiation may be a result of a negative feedback mechanism that compensates for the decreased enzymatic activity of PHDs due to the low oxygen level^28^. Proteins for HIF-2α, HIF-3α, and PHD3 were not detected by our Western blot system in either the pre- or post-heartbeat group.

To estimate whether changes in protein levels of HIF-1α, pVHL, and PHD2 are regulated by mRNA levels, we assessed relative mRNA expression levels of those genes that were obtained by microarray analysis comparing global gene expression between the pre- and post-heartbeat groups. The changes in mRNA expression of HIF-1α and VHL in the post-heartbeat group compared with the pre-heartbeat group were both 1.0 fold, whereas the mRNA expression of PHD2 was considerably increased by 2.1 fold (Fig. 4F), suggesting that changes in protein expression levels of HIF-1α and pVHL are not regulated by mRNA level, whereas PHD2 is regulated by its transcription level in the heart primordium after heartbeat initiation.

### Pathway enrichment analysis for evaluating biological and physiological pathways that are up-regulated in the heart primordium after heartbeat initiation

Next, we performed unbiased pathway enrichment analysis using data for global gene expression detected by a microarray to assess whether the glycolytic pathway that is regulated by HIF-1α is enriched among altered genes associated with pathways in energy metabolism in the heart primordium after heartbeat initiation.

**Figure 5:**
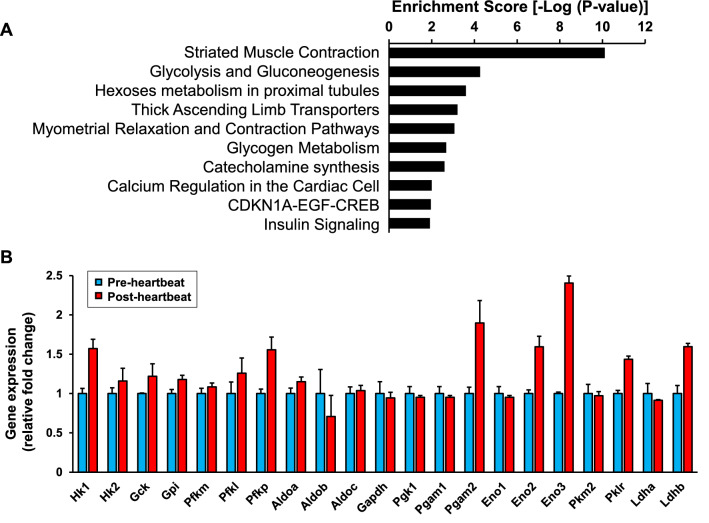
Pathway enrichment analysis for biological and physiological pathways that are up-regulated in the heart primordium after heartbeat initiation. (**A**) Top 10 pathways assessed by pathway enrichment analysis for biological and physiological pathways that were up-regulated by 1.5 fold or more in the post-heartbeat group compared with those in the pre-heartbeat group. (**B**) Individual values of relative gene expression of enzymes in glycolysis that are included in the pathway of “Glycolysis and Gluconeogenesis” in the pre- and post-heartbeat groups. Hk1 = Hexokinase-1, Hk2 = Hexokinase-2, Gck = Glucokinase, Gpi = Glucose-6-phosphate isomerase, Pfkm = 6-phosphofructokinase muscle type, Pfkl = 6-phosphofructokinase liver type, Pfkp = 6-phosphofructokinase platelet type, Aldoa = Aldolase A, Aldob = Aldolase B, Aldoc = Aldolase C, Gapdh = Glyceraldehyde-3-phosphate dehydrogenase, Pgk1 = Phosphoglycerate kinase 1, Pgam1 = Phosphoglycerate mutase 1, Pgam2 = Phosphoglycerate Mutase 2, Eno1 = Enolase 1, Eno2 = Enolase 2, Eno3 = Enolase 3, Pkm2 = Pyruvate kinase M2, Pklr = Pyruvate kinase L/R, Ldha = Lactate dehydrogenase A, Ldhb = Lactate Dehydrogenase B.

The enrichment scores in the top 10 enriched pathways among pathways listed in Wikipathways (http://www.wikipathways.org) for up-regulated genes by 1.5 fold or more in the post-heartbeat group are shown in Fig. 5. The best-matched pathway was the pathway of striated muscle contraction, being consistent with the grouping with pre-heartbeat and post-heartbeat initiation (Fig. 5A and Table 1). Notably, the pathway of glycolysis/gluconeogenesis ranked next to the best-matched pathway, whereas both of the pathways associated with the TCA cycle and mitochondrial OXPHOS were not found in the top ten enriched pathways (Fig. 5A). Since the beating heart is likely to catabolize glucose for ATP production, glycolysis but not gluconeogenesis is considered to be the most predominant pathway among metabolic pathways for up-regulated genes in the heart primordium after heartbeat initiation. The pathway of hexoses metabolism in proximal tubules, which ranked 3rd, includes some duplicated genes in the pathway of glycolysis/gluconeogenesis, suggesting that upregulation of genes of glycolysis is predominant after heartbeat initiation (Fig. 5A). Indeed, gene expression of major enzymes in glycolysis showed a considerable increase in the post-heartbeat group compared with that in the pre-heartbeat group (Fig. 5B). Taken together with the data obtained from metabolome analysis and the extracellular flux analyzer, predominantly up-regulated genes in glycolysis through HIF-1α are likely to underlie the increased glycolytic capacity leading to ATP production in the heart primordium after heartbeat initiation.

**Table 1:** Top 10 pathways that were up-regulated by 1.5 fold or more in the post-heartbeat group compared with those in the pre-heartbeat group.

Pathway	Matched entities	Total entities	%	p-value
Rn_Striated_Muscle_Contraction_WP316_87694	17	36	47.2	7.87E−11
Rn_Glycolysis_and_Gluconeogenesis_WP337_90443	6	42	14.3	5.41E−05
Rn_Hexoses_metabolism_in_proximal_tubules_WP3916_91332	6	55	10.9	2.51E−04
Rn_Thick_Ascending_Limb_Transporters_WP3882_91330	3	11	27.3	6.22E−04
Rn_Myometrial_Relaxation_and_Contraction_Pathways_WP140_87895	9	155	5.8	8.69E−04
Rn_Glycogen_Metabolism_WP160_89812	4	34	11.8	0.002100084
Rn_Catecholamine_synthesis_WP513_70129	2	5	40.0	0.002506614
Rn_Calcium_Regulation_in_the_Cardiac_Cell_WP326_72154	7	149	4.7	0.009949702
Rn_CDKN1A-EGF-CREB_WP2039_89165	5	94	5.3	0.011020469
Rn_Insulin_Signaling_WP439_69418	7	157	4.5	0.012211916

### Increased protein expression of HIF-1α downstream targets without changes in mitochondrial respiratory complexes in the heart primordium after heartbeat initiation

**Figure 6:**
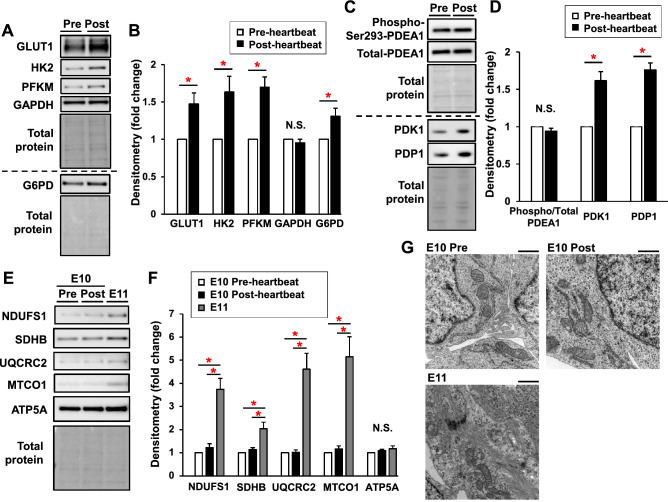
Increased protein expression of HIF-1α downstream targets without changes in mitochondrial respiratory complexes in the heart primordium after heartbeat initiation. (**A**,**B**) Representative images of Western blots (**A**) and densitometry (**C**) of GLUT1, HK2, PFKM, GAPDH, and G6PD in the pre- and post-heartbeat groups (N = 6). (**C**,**D**) Representative images of Western blots (**C**) and densitometry (**D**) of Ser 297 phospho-PDH E1α (PDEA1), total PDEA1, PDK1, and PDP1 in the pre- and the post-heartbeat groups (N = 6). (**E**,**F**) Representative images of Western blots (**E**) and densitometry (**F**) of NDUFS1, SDHB, UQCRC2, MTCO2, and ATP5A in the pre-heartbeat group, the post-heartbeat group, and the heart primordium at E11 (N = 6). (**G**) Representative transmission electron microscopy images of the heart primordium in the pre-heartbeat group, the post-heartbeat group, and the heart primordium at E11. Black scale bar indicates 1.0 μm. *p < 0.05 with Welch's t-test for comparison of two groups and ANOVA with Tukey–Kramer’s post hoc test for comparison of three groups. N.S.: not significant. Full-length blots/gels are presented in Supplementary Figures S2-S4.

We also assessed the expression of key enzymes regulated by the HIF-1 pathway in glycolysis, pentose phosphate pathway, and TCA cycles. Protein expression levels of transporter type 1 (GLUT1), hexokinase-2 (HK2), and 6-phosphofructokinase muscle type (PFKM), which have been reported to control glycolytic flux^29^, were significantly higher by 1.5 fold, 1.6 fold, and 1.7 fold, respectively, in the post-heartbeat group than in pre-heartbeat group, while expression levels of GAPDH were comparable in the two groups (Fig. 6A,B). The level of glucose-6-phosphate dehydrogenase (G6PD), which plays a role as a rate-limiting step in the pentose phosphate pathway, was also significantly higher in the post-heartbeat group than in the pre-heartbeat group (Fig. 6A, B). Since HIF-1α is known to inhibit the TCA cycle via phosphorylation of PDH E1α subunit induced by activation of PDK1^21^, we assessed the levels of expression of PDK1 and phosphorylation of PDH E1α subunit (Fig. 6C,D). As expected, the expression level of PDK1 was significantly higher in the post-heartbeat group than in the pre-heartbeat group; however, phosphorylation levels of PDH E1α subunit were comparable in the two groups (Fig. 6C,D). We also assessed expression levels of pyruvate dehydrogenase phosphatase catalytic subunit 1 (PDP1) since PDP1 phosphatases PDH E1α to activate TCA cycles and its activity is known to be regulated by cellular calcium concentration^30^. The level of PDP1 was significantly increased in the post-heartbeat group compared with that in the pre-heartbeat group (Fig. 6C,D). These findings suggest that the activity of PDH E1α was not suppressed in the post-heartbeat group despite an increased PDK1 level, presumably due to simultaneously increased PDP1 expression, and thereby the activity of the TCA cycle was not changed.

Finally, we evaluated expression levels of subunits of the mitochondrial OXPHOS complexes. There were no significant differences in expression levels of in subunits of complexes I–V between the pre- and post-heartbeat groups (Fig. 6E,F). Mitochondrial morphology in the heart primordium assessed by transmission electron microscopy was also similar in the pre- and post-heartbeat groups (Fig. 6G). These results are consistent with the results of metabolome analysis and results for mitochondrial function (Figs. 2 and 3). Since it has been demonstrated that activated mitochondrial respiration is important for maturation in undifferentiated cells^10–12^, we also investigated expression in subunits of mitochondrial OXPHOS and morphological features in the heart primordium after appearance of the linear heart tube. The heart primordium at E11 showed a dramatic increase in subunits of mitochondrial OXPHOS except for complex V (Fig. 6E,F) and tubulated mitochondria with distinct sarcomeres were observed in the heart primordium at E11 (Fig. 6G). These results suggest that mitochondrial maturation in the heart primordium is proceeded with development but is not involved in the initiation of heartbeat.

## Discussion

The initiation of heartbeat is essential for cardiogenesis as well as whole embryonic development. It has been widely recognized that glycolysis is a major source of energy in the early embryo^10–12^; however, it remained unclear how intracellular metabolism responds to cover increased energy demand in order to drive excitation–contraction coupling as well as cellular differentiation and proliferation in the heart primordium after heartbeat initiation in a low oxygen environment. Here, we showed that HIF-1α-mediated enhancement in glucose metabolism with upregulation of a glucose transporter and rate-limiting enzymes in glycolysis leads to ATP production in response to increased energy demand in the heart primordium after heartbeat initiation. Mitochondrial OXPHOS was not activated or upregulated at the timing of heartbeat initiation in the heart primordium, suggesting that mitochondrial maturation is not a main contributor to heartbeat initiation (Figs. 3C–F and 6E–G). In addition, enhanced glucose metabolism was accompanied by activation of the pentose phosphate pathway, potentially leading to antioxidant defense and nucleotide biosynthesis^25,31^ (Figs. 2B and 6A,B). Thus, our results showed how metabolic adaptation that is required for initiating heartbeat occurs in the heart primordium.

We proposed that activation of both glycolysis and the pentose phosphate pathway is responsible for the increased glucose carbon flow after heartbeat initiation because protein expression levels of HK2 and PFKM, which are rate-determining steps of glycolysis, were increased, and the protein level of G6PD, which is the rate-limiting step of the pentose phosphate pathway, was simultaneously increased after heartbeat initiation (Fig. 6A,B). The results of metabolome analysis showing that intermediate metabolites in glycolysis were decreased and GSH, a by-product of the pentose phosphate pathway, was increased after heartbeat initiation support our proposal (Fig. 2B). Activation of the pentose phosphate pathway after heartbeat initiation is considered to be reasonable since the pathway is associated with antioxidant defense and nucleotide biosynthesis, both of which are important for differentiation, proliferation, energy metabolism, and signal transduction in the beating and developing cells. Consistently, our metabolome analysis also suggests that total adenylate and total guanylate were considerably increased in the post-heartbeat group compared with that in the pre-heartbeat group, possibly reflecting increased biosynthesis of purine nucleotides with heartbeat (Fig. 2D–G). Although the significance of the pentose phosphate pathway in the embryonic heart primordium in the period around the initial heartbeat has not been investigated, a recent study by Miyazawa et al*.* showed that in the whole embryo at E8.5 to E10.5 in mice, corresponding to around E10.0 to E12.0 in rats^7^, presumably when the heart primordium already begins beating, the glucose carbon flow in glycolysis is changed into the pentose phosphate pathway with embryonic development via suppression of PFK and aldolase^32^. Activation of the pentose phosphate pathway after heartbeat initiation observed in the present study was similar to that study, but expression of PFKM was not decreased but rather increased (Fig. 6A,B). One plausible explanation for this difference is that the increase in energy demand associated with heartbeat may have required activation of the glycolytic pathway for ATP production in parallel with the pentose phosphate pathway. Nevertheless, activation of the pentose phosphate pathway in parallel with increased glycolysis is an important characteristic in the metabolic response at the timing of initial heartbeat. Furthermore, increased G6PD expression level in the beating heart primordium may contribute to not only the metabolic switch but also excitation–contraction coupling, in which G6PD was reported to increase Ca^2+^ current via interaction with the L-type calcium channel^33^.

Our findings suggest that the activated HIF-1α pathway is an upstream regulator in these metabolic changes after heartbeat initiation. It has been recognized that the HIF-1α pathway is not only a system in preparing for pathological hypoxia but also an important system for regulating physiological pathways in response to hypoxia by activating transcriptional upregulation of many genes associated with vasculogenesis, proliferation, and energy metabolism^16–18^. In fact, our microarray results showed that the gene expression of glycolysis/gluconeogenesis, in which HIF-1α is one of the major regulatory pathways, was preferentially upregulated in the heart primordium after heartbeat initiation compared with that before heartbeat initiation (Fig. 5A,B). In addition to gene expression in glycolysis/gluconeogenesis, HIF-1α has been reported to regulate genes with changes of phenotype in glycogen metabolism^34^, catecholamine synthesis^35^, and insulin signaling^36^. Our data showed that these pathways were exactly highlighted as enriched pathways for the beating heart primordium, supporting the idea that HIF-1α activation is an important upstream regulator for the multiple pathways for normal development in the heart primordium in the period of the initial heartbeat (Fig. 5A). Furthermore, considering the results of a study by Compernolle et al. showing that HIF-1α knockout embryos at E8.5 in mice, corresponding to around E10.0 in rats^7^, were not able to survive in a culture medium containing sufficient glucose even in a condition of normoxia^21^, it is likely that HIF-1α plays essential roles in not only glucose utilization but also cellular survival in the heart primordium after heartbeat initiation. For the mechanisms of HIF-1α activation with increased protein expression after heartbeat initiation (Fig. 4A–C), we hypothesize two regulation pathways: hypoxia-dependent regulation and hypoxia-independent regulation. As with the canonical HIF-1 pathway, hypoxia may have decreased the activity of PHD1-3, which hydroxylates proline residues on HIF-1α, and thereby reduced pVHL-mediated ubiquitination and degradation of HIF-1α in the heart primordium after heartbeat initiation, leading to increased expression and nuclear translocation of HIF-1α^15^. Given the fact that cardiac progenitor cells differentiate and proliferate with development, the increased number and density of cells may simply have contributed to the reduction in intracellular oxygen levels in the heart primordium after heartbeat initiation. However, our results showed that there was ATP synthesis-linked mitochondrial oxygen consumption in both the pre- and post-heartbeat groups and that it was increased with heartbeat initiation (Fig. 3A). This finding indicates the possibility that mitochondria in cardiac progenitor cells can respond to energy expenditure, that is, enhance breakdown of ATP to ADP, and can consume oxygen in the heart primordium in the period around initial heartbeat. Since measurement of the exact oxygen level in the heart primordium in a mammalian embryo being inside the uterus has been challenging, changes in oxygen levels in the heart primordium before and after heartbeat initiation were not evaluated in the present study. Nevertheless, a previous study revealed that hypoxic regions assessed by staining with a nitroimidazole-based hypoxia marker became widespread with development in the early embryonic stage and the heart was one of the strongest hypoxic tissues^37^, suggesting that the lower oxygen levels are actually involved in the activation of HIF-1α.

As for hypoxia-independent regulation of HIF-1α, our results showed that expression of pVHL was significantly decreased via posttranscriptional regulation in the heart primordium after heartbeat initiation (Fig. 4B,C,F). Reduction of the level of pVHL, which can negatively regulate HIF-1α independently of cellular oxygen level^38^, may have been associated with activation of HIF-1α after heartbeat initiation. Indeed, our finding that the expression level of PHD2, which can negatively regulate the protein level of HIF-1α, was rather increased in the heart primordium after heartbeat initiation supports the possibility of the presence of hypoxia-independent regulation of HIF-1α (Fig. 4D–F). The mechanism of the reduction of pVHL level after heartbeat initiation remains undetermined, although post-transcriptional regulation through ubiquitin E3 ligase^39,40^ or destabilization via ubiquitin-specific protease^41^ is a possibility. Interestingly, a recent study showed that phospholamban, an important regulator in calcium handling in the sarcoplasmic reticulum in cardiomyocytes, is downregulated by pVHL-mediated degradation in the failing heart^42^. Considering that study, the decreased level of pVHL may contribute to the maintenance of cellular calcium handling via regulation of phospholamban in the beating heart primordium.

There are several limitations in the present study. First, we distinguished the pre- and post-heartbeat groups based only on previous ex vivo studies^2,3,8,9^. Future innovations in in vivo imaging technology may reveal more detailed mechanisms in the alteration of energy metabolism in the heart primordium at more specific onset of heartbeat initiation. Second, the heart primordium was isolated from the body of the embryo without discriminating the first heart field (FHF) and second heart field (SHF). The difference in metabolic changes observed in the present study between the FHF and SHF after heartbeat initiation was not uncovered in the present study. Finally, we acknowledge that animal models of gene-editing or pharmacological intervention to determine causal relationships between the HIF-1 pathway and metabolic changes were not used in this study. However, to the best of our knowledge, there has been no previous report addressing characteristics of metabolic adaptation induced by heartbeat initiation in the heart primordium from the viewpoints of cellular physiology and metabolism. The present study included unbiased multi-omics approaches and functional metabolic measurements in isolated cells from the embryonic heart primordium as well as investigation of molecule expression, revealing the overall phenotype of metabolic changes after heartbeat initiation in the heart primordium. Further studies are needed to determine the master regulatory factor that controls the timing heartbeat initiation.

In summary, our results showed that enhanced glucose metabolism via activation of HIF-1α contributes to covering the increased energy demand in the heart primordium after heartbeat initiation. We also propose that concomitant activation of the pentose phosphate pathway via HIF-1α activation in the heart primordium after heartbeat initiation contributes to not only nucleotide biosynthesis but also increased production of reduced glutathione to prepare for increased oxidative stress. These metabolic adaptations may enable the maintenance of excitation–contraction coupling after heartbeat initiation in the heart primordium in a low oxygen environment. Our findings provide a novel association between the initiation of heartbeat and metabolic changes accompanied by activation of the HIF-1α pathway and may contribute to the future development of the research field in regenerative medicine in the heart.

## Materials and methods

### Ethics

The present study was approved by the Committee for Animal Research, Sapporo Medical University (19-010), and was performed in accordance with guidelines from the National Research Council of the National Academies, USA. In addition, the study was carried out in compliance with the ARRIVE (Animal Research: Reporting of In Vivo Experiments) guidelines.

### Animal preparation

Wistar rats were bred in a dark–light reversal room (lights off at 4:00 am–4:00 pm). Mating was performed by placing one female rat and two male rats in the same cage at 9:00 am until the end of one dark period. Embryonic day 0.00 (E 0.00) was defined as the mid-point of the previous dark period (10:00 am) when a vaginal plug was confirmed in the next light cycle. Since we previously reported that the embryonic heart primordia of Wistar rats begin to contract at E9.99–E10.13^6^, pregnant rats were sacrificed from 9:30 am (at E9.98) to 1:00 pm (at E10.13) to obtain embryos before and after heartbeat initiation. Pregnant rats were sacrificed by carbon dioxide euthanasia. After euthanasia of the mother had been confirmed by respiratory arrest and discoloration of the pupil, the abdomen of the mother was immediately opened, and the embryos with the uterus were quickly removed from the body and were placed in a small incubator (MI-IBC, Olympus, Tokyo, Japan) containing Tyrode solution (143 mM NaCl, 5.4 mM KCl, 0.5 mM MgCl_2_, 1.8 mM CaCl_2_, 0.33 mM NaH_2_PO_4_, 5.5 mM glucose, and 5.0 mM HEPES, pH adjusted to 7.4 with NaOH) on ice or at room temperature, depending on the following experiments. The embryos were isolated from the uterus and heart primordia were quickly and carefully excised from the bodies of embryos under a microscope in the incubator, and the isolated heart primordia were consecutively used or were immediately frozen in liquid nitrogen and stored at −80 °C until the day of experiments, depending on the kinds of experiments.

### Metabolome analysis

Six pooled embryonic heart primordia were homogenized with ice-cold phosphate-buffered saline with 0.1% Triton-X and proteinase inhibitor cocktail (Promega, Madison, WI) and the lysate was centrifuged at 3,000 g for 10 min at 4 °C to obtain the supernatant. The protein concentration in the lysate was measured by the BCA assay (Takara-bio, Japan) and was adjusted to 0.5 μg/μl. Metabolome analysis in the tissue lysate was performed by capillary electrophoresis time-of-flight mass spectrometry (CE-TOFMS) and capillary electrophoresis tandem mass spectrometry (CE-MS/MS) in the faculty of Human Metabolome Technologies Inc. as previously described^43,44^. In brief, the lysate containing heart primordium extract was supplemented with 50% acetonitrile/Milli-Q water containing internal standards. Following centrifugation of the lysate at 2,300 g for 5 min, the upper aqueous layer was centrifugally filtered via a Millipore 5-kDa cutoff filter. The filtrate was then centrifugally concentrated and re-suspended in Milli-Q water. Samples were diluted 1:2 (v/v) by Milli-Q water for measurement of cationic compounds and were diluted for 1:5 (v/v) by Milli-Q water for the measurement of anionic compounds. Cationic compounds were measured in the positive mode of CE-TOFMS and anionic compounds were measured in the positive and negative modes of CE-MS/MS. Peaks were automatically detected and were analyzed using the following software: MasterHands (Keio University, Tsuruoka, Japan) and MassHunter Quantitative Analysis B.06.00 (Agilent Technologies, Santa Clara, CA, USA), respectively. Concentrations of metabolites were calculated by normalizing the peak area of each metabolite with respect to the area of the internal standard. Principal component analysis was performed by in-house statistical software developed by Human Metabolome Technologies Inc. As a preprocessing of the data in these analyses, eps (≈0) were substituted for metabolites that were below the detection sensitivity in some samples and standardization (µ = 0, σ = 1) was carried out.

### Glycogen content

Glycogen content in the heart primordium was measured using Glycogen Colorimetric Assay Kit II (BioVision, Japan) since glycogen content was not assessed in the metabolome analysis.

### Measurement of mitochondrial and glycolytic functions

Oxygen consumption rate (OCR) and extracellular acidification rate (ECAR) were measured by using Seahorse XFe96 Bioanalyzer (Agilent Technologies) as previously described with slight modifications^45^. The embryonic heart primordia were incubated with Ca^2+^-free Tyrode solution containing 25 mg/ml collagenase (Wako Chemicals, Osaka, Japan) for 30 min at 37 °C to obtain freshly isolated single cells as previously demonstrated^46^. After collecting cells by the centrifugation at 2000 g, 10 × 10^3^ isolated cells in Ca^2+^-free Tyrode solution were placed in each well of a 96-well assay plate. Subsequently, the plate was centrifuged at 2000 g for 10 min. Following centrifugation, the Tyrode solution was replaced with Seahorse XF DMEM assay medium (Agilent Technologies) containing 5.5 mM glucose, 1.0 mM pyruvate and 5.0 mM HEPES (pH adjusted 7.4 with NaOH), and OCR and ECAR were measured in a Seahorse XFe96 Bioanalyzer at baseline and following injections of 5.0 μM oligomycin, 5.0 μM carbonyl cyanide p-trifluoromethoxyphenylhydrazone (FCCP), and 5.0 μM rotenone and antimycin A. Values of OCR and ECAR were normalized to the amount of protein per well.

### Microarray analysis

Microarray analysis for global gene expression was performed using G3 Rat GE 8 × 60 K (Agilent Technologies) by Hokkaido System Science according to the manufacturer's guide. In brief, total RNA was extracted from the embryonic heart primordium using an RNeasy micro Kit (Qiagen). After the quality of RNA had been verified, Cyanie3-labeled cRNA was generated using a Low Input Quick Amp Labeling Kit (Agilent Technologies) and was applied onto a microarray. Following fragmentation and hybridization at 65 °C for 17 h, the microarray was scanned using an Agilent Technologies Microarray Scanner and the intensity of signals in each spot was quantified by Agilent Feature Extraction software. Analysis of the microarray data was performed using GeneSpring software version 13.1.1. Data normalization was performed as a 75th percentile shift, and baseline transformation was performed as a median of all samples. Pathway analysis through Wikipathways (http://www.wikipathways.org) was performed using the Single Enrichment Analysis tool in GeneSpring software. Genes that were upregulated by 1.5 fold or more and p values assessed by the Tukey HSD test of less than 0.05 in the post-heartbeat group compared to those in the pre-heartbeat group were selected for pathway analysis. The datasets are available in the Gene Expression Omnibus (GEO) database (accession number: GSE185702).

### HIF-1α DNA-binding activity

An HIF-1α Transcription Factor Assay Kit (Abcam) was used to evaluate DNA-binding activity of HIF-1α in the nuclear fraction of the heart primordium according to the manufacturer’s instructions. The nuclear fraction was obtained by using a Nuclear Extraction Kit (Abcam).

### Western blots

Embryonic heart primordia were homogenized in ice-cold buffer (CelLytic™ Cell Lysis Reagent, Sigma-Ardrich) containing a protease inhibitor (Promega) and a phosphatase inhibitor cocktail (PhosSTOP, Roche Molecular Biochemicals). To obtain the total homogenate, the lysate was centrifuged at 3000 g for 10 min at 4 °C and the supernatant was collected. Protein concentration was determined using the Qubit Protein Assay Kit (Invitrogen, Carlsbad, CA). Equal amounts of proteins were electrophoresed on 4–12% Bis–Tris polyacrylamide gels (Life Technologies, Carlsbad, CA) and transferred to a nitrocellulose membrane. (Invitrogen). Total proteins on the membrane were visualized by 0.1% (w/v) of Ponceau S in 5% acetic acid. After being blocked with Tris Buffer Saline containing 5% skimmed milk and 0.05% Tween 20, the membrane was incubated overnight with primary antibodies against HIF-1α (CST #14,179, Cell Signaling, Danvers, MA), HIF-1β/ARNT (CST #5537S, Cell Signaling), PHD1 (ab113077, Abcam, Cambridge, MA), pVHL (sc-135657, Santa Cruz, Dallas, TX), PHD2 (CST #3293, Cell Signaling), GLUT1 (ab115730, Abcam), HK2 (sc-374091, Santa Cruz), PFKM (ab154804, Abcam), and GAPDH (sc-25778, Santa Cruz) for the glycolytic pathway, G6PD (ab76598, Abcam) for the pentose phosphate pathway, phospho-PDH E1α subunit Ser 297 (ab177461, Abcam), total PDH E1α subunit (ab110330, Abcam), PDK1 (LS‑B1733, LSBio, Seattle, WA), and PDP1 (sc-398117, Santa Cruz) for the TCA cycle, and Total OXPHOS Rodent WB Antibody Cocktail (ab110413, Abcam) containing targets for NDUFB8, SDHB, UQCRC2, MTCO2, and ATP5A for mitochondrial respiratory chain subunits. Since the bands for NDUF8 were faint, an antibody with NDUFS1 (sc-271387, Santa Cruz) was used for evaluating complex I instead. Immunoblotted proteins were visualized by an Immobilon Western Detection Kit (Millipore, Billerica, MA). Images of the membrane were developed, photographed, and processed using a ChemiDoc XRS + System with Image Lab software (Bio-Rad, Hercules, CA). The proteins were quantified by measuring intensities of individual bands by Image J software (National Institutes of Health).

### Transmission electron microscopy

The heart primordium isolated from the whole embryo was fixed in 2.5% glutaraldehyde and 0.1 M sodium cacodylate buffer at 4 °C overnight, followed by treatment with 1% osmium tetroxide in 0.1 M sodium cacodylate. After dehydration with ethanol and propylene oxide, the samples were embedded in the mold of an epoxy resin block and cut with a microtome. Finally, the samples were electronically stained with uranyl acetate and lead citrate and examined with an electron microscope (H7650, Hitachi, Japan).

### Statistics

Data are expressed as means ± SEM. For sample size estimation, we aimed to detect ~ 20% difference in the experiments including glycogen content, mitochondrial and glycolytic functions, HIF-1α DNA-binding activity assay, and Western blots. Given an estimated standard deviation of ~ 15% in each group, we estimated five to six samples would be needed for statistically comparing groups. Differences between two groups were statistically analyzed using Welch's t-test. Differences among three groups were assessed by one-way analysis of variance (ANOVA), and the Tukey–Kramer’s post hoc test was used for multiple comparisons when ANOVA indicated significant differences. A P value less than 0.05 was considered statistically significant for these tests. For the multi-omics experiments including metabolome analysis and microarray analysis, statistical analyses were performed using the methods as described in each section. Individual values that were obtained from these multi-omics experiments were simply represented as means and SEM in order to avoid the limitations of small sample sizes, multiple testing, and data extraction errors.

## Supplementary Information

Supplementary Information 1.

Supplementary Information 2.
