# Supplementary material for: Enhanced glucose metabolism through activation of HIF-1α covers the energy demand in a rat embryonic heart primordium after heartbeat initiation
Source: Sci Rep. 2022 Jan 7;12:74. doi: 10.1038/s41598-021-03832-5 (PMC8741773; doi:10.1038/s41598-021-03832-5)
Supplement: Supplementary file 1 — Supplementary Information 1. [file 41598_2021_3832_MOESM1_ESM.pptx]

## Slide 1
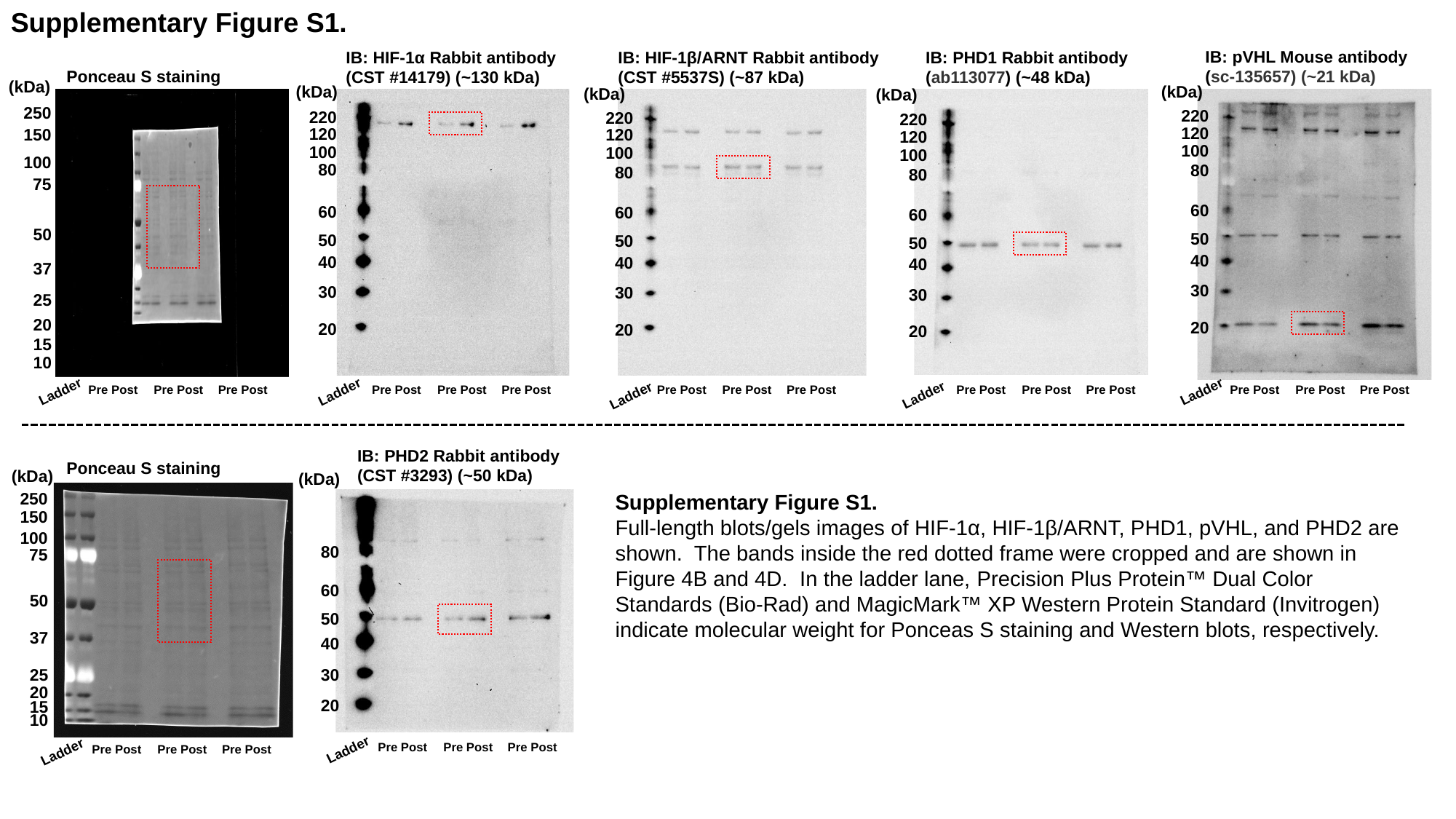

Supplementary Figure S1.
IB: pVHL Mouse antibody
(sc-135657) (~21 kDa)
IB: PHD1 Rabbit antibody
(ab113077) (~48 kDa)
IB: HIF-1β/ARNT Rabbit antibody
(CST #5537S) (~87 kDa)
IB: HIF-1α Rabbit antibody
(CST #14179) (~130 kDa)
Ponceau S staining
(kDa)
(kDa)
(kDa)
(kDa)
(kDa)
250
220
220
220
220
120
120
150
120
120
100
100
100
100
100
80
80
80
80
75
60
60
60
60
50
50
50
50
50
40
40
40
40
37
30
30
30
30
25
20
20
20
20
20
15
10
Pre Post
Pre Post
Pre Post
Pre Post
Pre Post
Pre Post
Pre Post
Pre Post
Pre Post
Pre Post
Pre Post
Pre Post
Pre Post
Pre Post
Pre Post
Ladder
Ladder
Ladder
Ladder
Ladder
IB: PHD2 Rabbit antibody
(CST #3293) (~50 kDa)
Ponceau S staining
(kDa)
(kDa)
250
Supplementary Figure S1.
Full-length blots/gels images of HIF-1α, HIF-1β/ARNT, PHD1, pVHL, and PHD2 are shown. The bands inside the red dotted frame were cropped and are shown in Figure 4B and 4D. In the ladder lane, Precision Plus Protein™ Dual Color Standards (Bio-Rad) and MagicMark™ XP Western Protein Standard (Invitrogen) indicate molecular weight for Ponceas S staining and Western blots, respectively.
150
100
80
75
60
50
50
37
40
25
30
20
20
15
10
Pre Post
Pre Post
Pre Post
Ladder
Pre Post
Pre Post
Pre Post
Ladder

## Slide 2
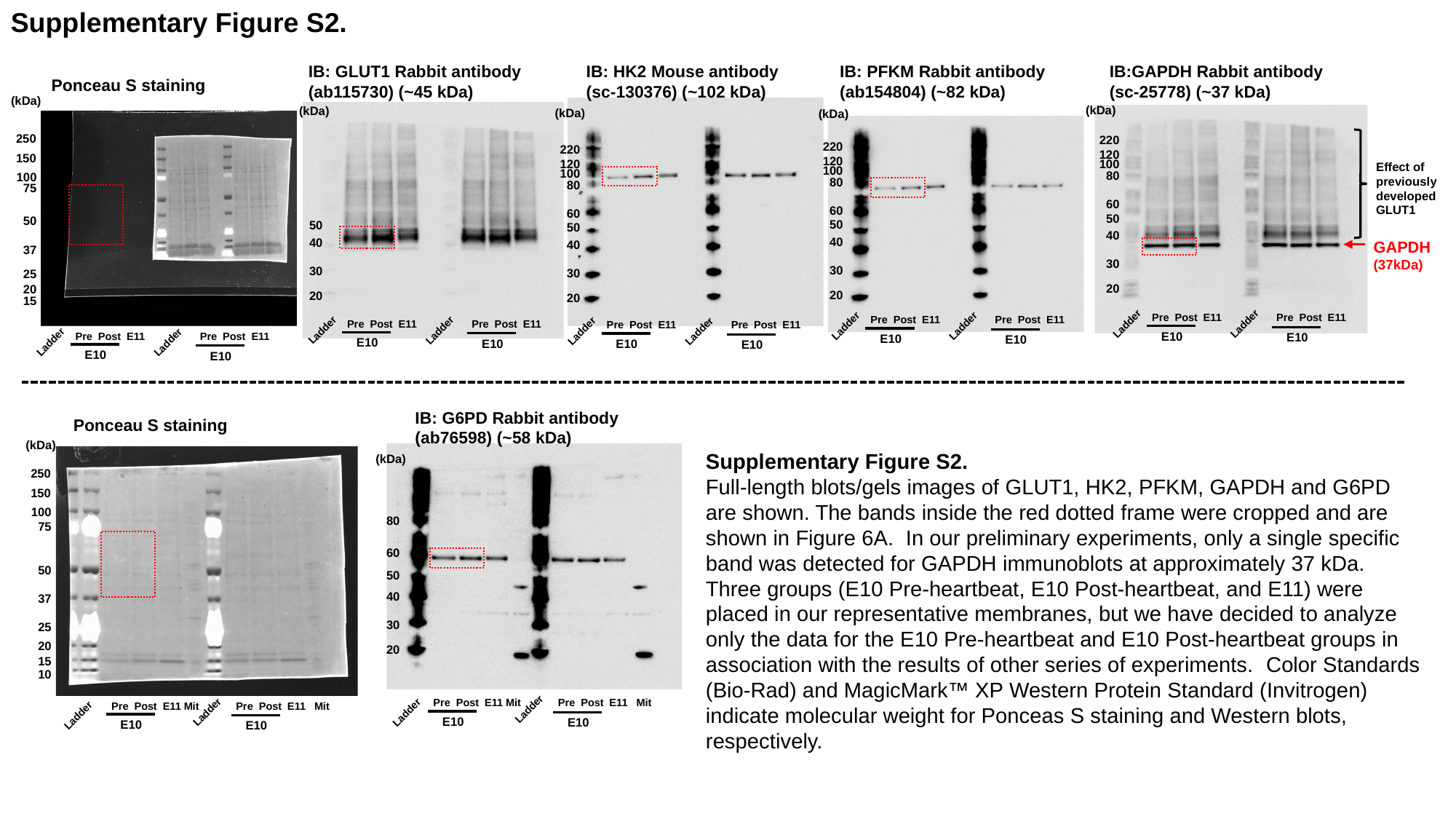

Supplementary Figure S2.
IB: GLUT1 Rabbit antibody
(ab115730) (~45 kDa)
IB: HK2 Mouse antibody
(sc-130376) (~102 kDa)
IB: PFKM Rabbit antibody
(ab154804) (~82 kDa)
IB:GAPDH Rabbit antibody
(sc-25778) (~37 kDa)
Ponceau S staining
(kDa)
(kDa)
(kDa)
(kDa)
(kDa)
250
220
220
220
120
150
120
120
100
Effect of
previously
developed
GLUT1
100
100
80
100
80
80
75
60
60
60
50
50
50
50
50
40
40
40
GAPDH
(37kDa)
40
37
30
30
30
30
25
20
20
20
20
20
15
Pre Post E11
Pre Post E11
Pre Post E11
Pre Post E11
Ladder
Ladder
Pre Post E11
Pre Post E11
Pre Post E11
Pre Post E11
Ladder
Ladder
Ladder
Ladder
Ladder
Ladder
E10
Pre Post E11
Pre Post E11
E10
E10
E10
E10
Ladder
Ladder
E10
E10
E10
E10
E10
IB: G6PD Rabbit antibody
(ab76598) (~58 kDa)
Ponceau S staining
(kDa)
Supplementary Figure S2.
Full-length blots/gels images of GLUT1, HK2, PFKM, GAPDH and G6PD are shown. The bands inside the red dotted frame were cropped and are shown in Figure 6A. In our preliminary experiments, only a single specific band was detected for GAPDH immunoblots at approximately 37 kDa. Three groups (E10 Pre-heartbeat, E10 Post-heartbeat, and E11) were placed in our representative membranes, but we have decided to analyze only the data for the E10 Pre-heartbeat and E10 Post-heartbeat groups in association with the results of other series of experiments. Color Standards (Bio-Rad) and MagicMark™ XP Western Protein Standard (Invitrogen) indicate molecular weight for Ponceas S staining and Western blots, respectively.
(kDa)
250
150
100
80
75
60
50
50
40
37
30
25
20
20
15
10
Pre Post E11 Mit
Pre Post E11 Mit
Pre Post E11 Mit
Pre Post E11 Mit
Ladder
Ladder
Ladder
Ladder
E10
E10
E10
E10

## Slide 3
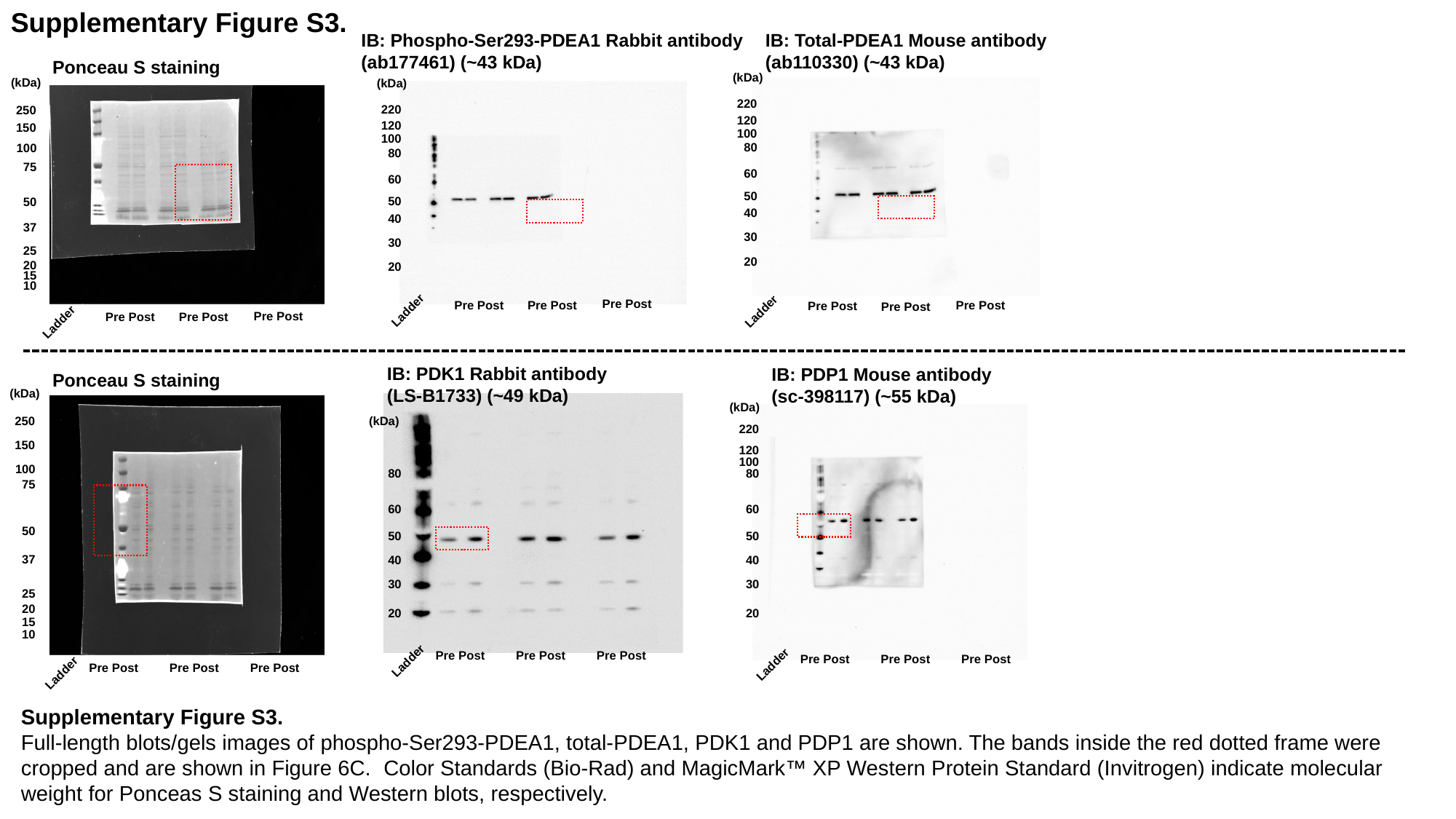

Supplementary Figure S3.
IB: Phospho-Ser293-PDEA1 Rabbit antibody
(ab177461) (~43 kDa)
IB: Total-PDEA1 Mouse antibody
(ab110330) (~43 kDa)
Ponceau S staining
(kDa)
(kDa)
(kDa)
220
220
250
120
120
150
100
100
(kDa)
80
100
80
75
60
60
50
50
50
40
40
37
30
30
25
20
20
20
15
10
Pre Post
Pre Post
Pre Post
Pre Post
Pre Post
Pre Post
Ladder
Ladder
Pre Post
Pre Post
Pre Post
Ladder
IB: PDK1 Rabbit antibody
(LS‑B1733) (~49 kDa)
IB: PDP1 Mouse antibody
(sc-398117) (~55 kDa)
Ponceau S staining
(kDa)
(kDa)
(kDa)
250
220
150
120
100
100
80
80
75
60
60
50
50
50
37
40
40
30
30
25
20
20
20
15
10
Pre Post
Pre Post
Pre Post
Pre Post
Pre Post
Pre Post
Ladder
Ladder
Pre Post
Pre Post
Pre Post
Ladder
Supplementary Figure S3.
Full-length blots/gels images of phospho-Ser293-PDEA1, total-PDEA1, PDK1 and PDP1 are shown. The bands inside the red dotted frame were cropped and are shown in Figure 6C. Color Standards (Bio-Rad) and MagicMark™ XP Western Protein Standard (Invitrogen) indicate molecular weight for Ponceas S staining and Western blots, respectively.

## Slide 4
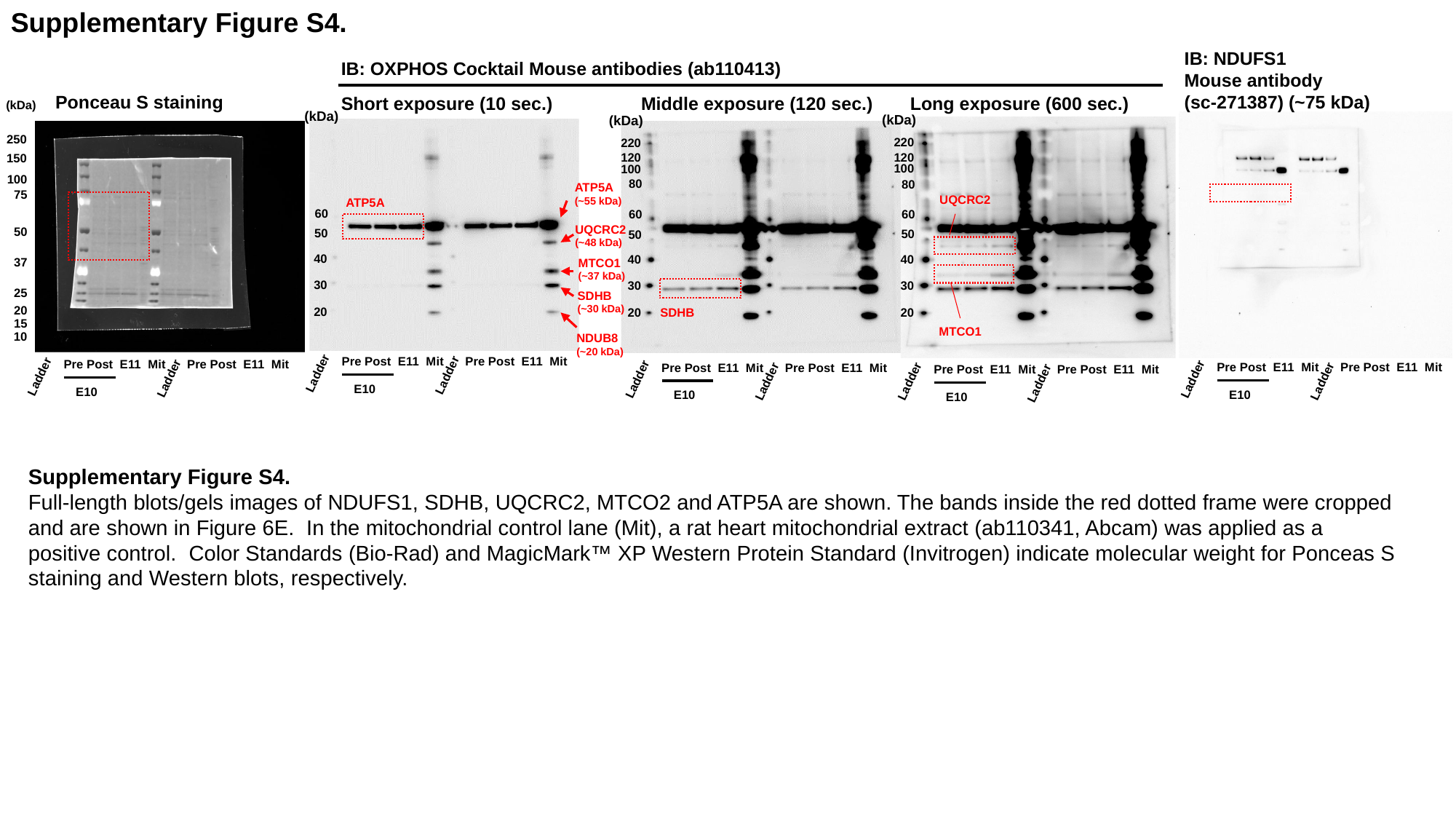

Supplementary Figure S4.
IB: NDUFS1
Mouse antibody
(sc-271387) (~75 kDa)
IB: OXPHOS Cocktail Mouse antibodies (ab110413)
Ponceau S staining
Short exposure (10 sec.)
Middle exposure (120 sec.)
Long exposure (600 sec.)
(kDa)
(kDa)
(kDa)
(kDa)
250
220
220
120
120
150
100
100
100
80
80
ATP5A
(~55 kDa)
75
UQCRC2
ATP5A
60
60
60
UQCRC2
(~48 kDa)
50
50
50
50
40
40
40
37
MTCO1
(~37 kDa)
30
30
30
25
SDHB
(~30 kDa)
20
20
20
20
SDHB
15
MTCO1
10
NDUB8
(~20 kDa)
Pre Post E11 Mit
Pre Post E11 Mit
Pre Post E11 Mit
Pre Post E11 Mit
Pre Post E11 Mit
Pre Post E11 Mit
Pre Post E11 Mit
Pre Post E11 Mit
Pre Post E11 Mit
Pre Post E11 Mit
Ladder
Ladder
Ladder
Ladder
Ladder
Ladder
Ladder
Ladder
Ladder
Ladder
E10
E10
E10
E10
E10
Supplementary Figure S4.
Full-length blots/gels images of NDUFS1, SDHB, UQCRC2, MTCO2 and ATP5A are shown. The bands inside the red dotted frame were cropped and are shown in Figure 6E. In the mitochondrial control lane (Mit), a rat heart mitochondrial extract (ab110341, Abcam) was applied as a positive control. Color Standards (Bio-Rad) and MagicMark™ XP Western Protein Standard (Invitrogen) indicate molecular weight for Ponceas S staining and Western blots, respectively.
